# Social networks and secondary health conditions: The critical secondary team for individuals with spinal cord injury

**DOI:** 10.1179/2045772312Y.0000000035

**Published:** 2012-09

**Authors:** Sara J. T. Guilcher, Tiziana Casciaro, Louise Lemieux-Charles, Catharine Craven, Mary Ann McColl, Susan B. Jaglal

**Affiliations:** 1 Institute of Health, Policy, Management & Evaluation, University of Toronto, Toronto, Ontario, Canada; 2 Rotman School of Management, University of Toronto, Toronto, Ontario, Canada; 3 Institute of Health, Policy, Management & Evaluation, University of Toronto, Toronto, Ontario, Canada; Toronto Rehabilitation Institute, University Health Network, Toronto, Ontario, Canada; and Department of Medicine, University of Toronto, Toronto, Ontario, Canada; 4 Centre for Health Services and Policy Research, Queen's University, Kingston, Ontario, Canada; and Department of Community Health and Epidemiology and School of Rehabilitaion Therapy, Queen's University, Kingston, Ontario, Canada; 5 Institute of Health, Policy, Management & Evaluation, University of Toronto, Toronto, Ontario, Canada; Toronto Rehabilitation Institute, University Health Network, Toronto, Ontario, Canada; Graduate Department of Rehabilitation Science, University of Toronto, Toronto, Ontario, Canada; and Department of Physical Therapy, University of Toronto, Toronto, Ontario, Canada

**Keywords:** Social support, Health care, Community networks, Spinal cord injuries, Secondary complications

## Abstract

**Objectives:**

To describe the structure of informal networks for individuals with spinal cord injury (SCI) living in the community, to understand the quality of relationship of informal networks, and to understand the role of informal networks in the prevention and management of secondary health conditions (SHCs).

**Design:**

Mixed-method descriptive study.

**Setting:**

Ontario, Canada

**Participants:**

Community-dwelling adults with an SCI living in Ontario

**Interventions/methods:**

The Arizona Social Support Interview Survey was used to measure social networks. Participants were asked the following open-ended questions: (1) What have been your experiences with your health care in the community? (2) What have been your experiences with care related to prevention and/or management of SHCs?, (3)What has been the role of your informal social networks (friends/family) related to SHCs?

**Results:**

Fourteen key informant interviews were conducted (6 men, 8 women). The overall median for available informal networks was 11.0 persons (range 3–19). The informal network engaged in the following roles: (1) advice/validating concerns; (2) knowledge brokers; (3) advocacy; (4) preventing SHCs; (5) assisting with finances; and (6) managing SHCs. Participants described their informal networks as a “secondary team”; a critical and essential force in dealing with SHCs.

**Conclusions:**

While networks are smaller for persons with SCI compared with the general population, these ties seems to be strong, which is essential when the roles involve a level of trust, certainty, tacit knowledge, and flexibility. These informal networks serve as essential key players in filling the gaps that exist within the formal health care system.

## Introduction

Spinal cord injury (SCI) involves significant change(s) with motor, sensory, and/or autonomic functioning, and is associated with high levels of disability.^1^ In Canada, approximately 44 000 individuals currently live with traumatic SCI^1,2^ and approximately 1100 new cases occur each year,^3,4^ whereas higher incidence and prevalence rates are reported in the United States of America (USA) with 12 400 new cases per year and an estimated 259 000 prevalent cases.^5^ Advances in roadside management, early acute medical therapy and surgical decompression, and advances in rehabilitation care have contributed to increased life-expectancy and frequency of community discharge^6^ with the mean survival time reported to be more than 30 years.^7^

While more individuals are surviving initial injury, they are predisposed to multi-system impairments that can lead to the development of serious secondary health complications (SHCs). These include respiratory impairments and related infections, urinary tract infections (UTIs), respiratory infections, heart disease, osteoporosis, upper extremity overuse injuries, sleep disorders, sexual disorders such as erectile dysfunction and ejaculation among men, pressure ulcers, chronic pain, fatigue, depression, and suicide.^8,9^ While many of these SHCs are preventable and/or responsive to appropriate primary care management,^9^ they are purported to be key contributors for re-hospitalization and/or death in the post-acute phase.^10–13^

These high utilization rates of health care services^11,14–17^ suggest that care needs in the community are not being met for this population. Given the reduced lengths of inpatient rehabilitation stay, persons with SCI often require outpatient community services to manage SHCs that have not stabilized at the time of index discharge.^18^

The shift from centralized to regionalized care has increased responsibility to individuals and their informal care networks, especially for those who are most vulnerable to navigating the health care system.^19,20^ Formal care providers are usually provided by paid medically trained professionals such as physicians, physical therapists, occupational therapists, nurses, speech language pathologists, social workers, psychologists, and personal attendants. Informal care providers are typically unpaid individuals with minimal previous formal training in health care.^21^ In contrast to formal providers, informal care providers typically have a pre-existing relationship with the individual for whom the care is being provided. Informal care can involve tasks such as management (organization and referrals), advocacy of care, assistance with cooking, shopping, cleaning, household maintenance, mobility, community participation, basic daily grooming, advice, and emotional support.^21^

Despite this shift in health service delivery to the community, to date there is a gap in the SCI literature with respect to understanding the formal and informal care-giving networks as they relate to the prevention of SHCs. Understanding the extent to which social systems influence health is just as critical as examining the more bio-medical risk factors of illness.^22^ Social capital, defined as “features of social organization, such as trust, norms, and networks that can improve efficiency of society by facilitating coordination and cooperation for mutual benefit”(p. 66),^23^ is an important construct in understanding social context. Broadly, social capital is a multi-faceted construct that relates to social relationships and the resources obtained through these relationships.^24^

Social networks are a key building block to social capital.^25,26^ Studying social networks, both formal and informal networks of care, as well as the pattern of their interactions, has been useful in understanding fragmentation of care in other populations with chronic conditions, such as mental health, who have high health care utilization.^27–33^ For example, integration of mental health services in the community has been challenging, as reflected by the “revolving door” phenomenon.^34^ This revolving-door concept is an indicator of fragmented care and refers specifically to bounce-back situations whereby patients have four or more admissions to inpatient services within a short time period (i.e. often a year or two).^35^

There is evidence to suggest that mental health care-giving networks can influence mental health care utilization and negative mental health outcomes.^27–32^ In particular, social capital measures such as network size and function have been suggested to influence mental health care use.^28,29,32^ For example, Pescosolido *et al.*^28,29^ investigated formal and informal networks and patterns of mental health care use for low income Puerto Ricans with mental health problems. Individuals with larger and more supportive informal networks of care had decreased visits to formal mental health providers.

Similarly, Bonin *et al.*,^32^ using the social network theory, examined mental health utilization among homeless individuals living in Quebec. These researchers were interested in examining factors that influenced health care use among those who were impoverished with a mental health disorder in a universal health care system. With the exception of illness history, Bonin *et al.*^32^ identified that social networks, environmental characteristics, and patient demographics all significantly predicted utilization of mental health services.

Bonin *et al.*'s^32^ findings suggest that the application of the social network theory to identify factors associated with high health care use may be beneficial for studying other vulnerable populations with chronic care needs, such as those with SCI. Understanding these dynamic interconnected factors such as the structure of care networks, the linkages within and between networks, and their overall function, especially for complex populations that interact frequently with the health care environment are important as a means to improve integration of care.^28,29,33,36^ This methodology has been useful in understanding fragmentation of care such that recommendations to improve the integration of care for the SCI population at the individual, provider, and policy level can then be made.

### Implications of research

We currently know that individuals with SCI have significant secondary complications and high health care utilization. SHCs continue to be problematic in approximately 20% of this population^37^ and with more than 50% self-reporting spasticity, pain, bladder infections in the past year, and for several SHCs including pressure ulcers and autonomic dysreflexia, the odds of developing these SHCs increased per year post injury.^9^ Despite the relatively low prevalence of SCI, the burdens imposed on the individual and health care system are significant, as demonstrated by high health care utilization, decreased quality of life, and considerable financial costs.^2,38^

Recently, a systematic review was conducted examining the role of social support and social skills for persons with SCI.^39^ The authors concluded that, in general, social support led to overall better health and functioning for persons with SCI.^39^ Thus, as this review highlights, understanding care provision and social networks in the community is important. While we know that SHCs are likely influencing health care use, we do not know what community factors are associated with these SHCs.

This study aimed to provide comprehensive descriptive analyses of community networks for community-dwelling individuals with SCI. This approach will highlight informal network characteristics and how networks influence the journey of care, defined as a complex series of interactions that comprise the processes of health care, as it relates to SHC management.^40,41^

### Objectives

Specifically, this study will:

1. describe the structure of informal networks (e.g., size and type of care providers) for individuals with SCI living in the community; and
2. understand the quality of relationships of informal networks
3. understand the role of informal networks in the prevention and management of SHCs.

## Methods

We used a mixed method descriptive approach.^42^ In-depth semi-structured interviews with community-dwelling individuals with an SCI were conducted. Owing to geographical and accessibility limitations, the interviews were conducted over the telephone and audio-recorded. The consumer interviews ranged from 60 to 90 minutes in length.

### Conceptual framework: Network Episode Model

**Figure 1:**
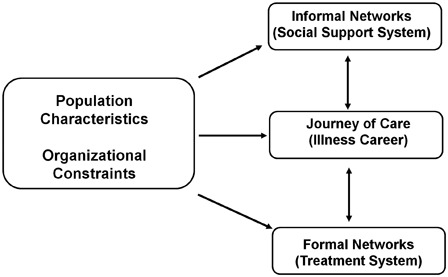
Conceptual framework based on the Network Episode Model

We used Pescosolido's (1991) Network Episode Model (NEM) as a conceptual guide for this study (see Fig. 1),^40^ as this model acknowledges the *interdependency* and social *context* that exists between individuals, networks, and their journeys of health care. The NEM has four domains, social context (environment), social support system (informal networks), the treatment system (formal networks) and the illness career (journey of care). Based on the social network theory, the NEM highlights the importance of community network *structures*, *processes*, and related *functional outcomes* as dynamic components that influence health behaviors and health outcomes.^40^ There are four underlying assumptions to this model, (1) communities contain care providers; (2) process of care is dynamic, occurs over time, and develops into patterns and pathways; (3) underlying the processes of health care use are social networks; and (4) social networks may influence the interaction with the care providers.^40^

While the NEM does not negate the role of the individual as an active agent, it recognizes that social influences can be as equally, if not more important.^40^ For the purpose of the present exploratory study, we focused specifically on the “population characteristics” and “informal networks” domains of the NEM. We focused in depth on these two domains given the time intensive nature of measuring social networks and efforts to reduce responder burden.

#### Theoretical position

The theoretical approach underlying this study was that of relativist ontology, that is, previous *a priori* knowledge helped inform assumptions but allowed for emerging themes to arise.^43^ The paradigm guiding this research question was a naturalistic interpretive one. This multi-lens approach was concerned with understanding the subjective, complex and contextual experiences of participants.^44^ The contextual and constructed realities of each participant helped inform and reshape knowledge gained from the research inquiry.^45^ Furthermore, principles from Thorne's interpretive description methodology facilitated the scientific inquiry, as this approach allowed for *a priori* assumptions (e.g. network episode theory) to be synthesized with knowledge gained from data, as well as other theoretical and contextual health services clinical knowledge.^45^

### Key informant recruitment

The recruitment strategy included purposeful snowball sampling for maximum variation in key informant experiences.^46^ We specifically aimed to have fair representation across gender, level of injury (cervical, thoracic, and lumbar), and mechanism of injury (traumatic and non-traumatic), as well as socioeconomic status/funding source for health care services (private payments from motor vehicle accident compensation and public payment for services). We recruited from the community by advertising the study via the Canadian Paraplegic Association (CPA)-Ontario division's website and email distribution. Semi-structured key informant interviews with community-dwelling individuals with an SCI provided the primary source of data. Key informants were at least 18 years of age as we focused specifically on adult experiences.

### Informed consent

Approval for this study was obtained from the University Health Network Research Ethics Board, as well as the University of Toronto. All participants provided informed consent prior to the interview.

### Data collection

Key informant interviews with participants were conducted using a semi-structured interview guide composed of a number of valid and reliable scales, open-ended questions and potential probes (see Table 1 for open-ended questions). Based on the NEM, a number of quantitative scales were used to describe socio-demographics and clinical characteristics of participants (items based on the Canadian Community Health Survey (CCHS, version 3.1)^47^ and the Ontario Spinal Cord Injury Registry (OSCIR),^48^ social networks (based on CCHS and the Arizona Social Support Interview Schedule (ASSIS)),^49^ and history of SHCs within the past year (Spinal Cord Injury Secondary Complications Scale (SCI-SCS)).^50^ The SCI-SCS was instrumental as a probe for detailed discussion related to how each of the identified SHCs was managed and the role informal networks played in these SHCs.

**Table 1:** Interview guide for open-ended questions with key informants*

1	What have been your experiences with your health care in the community?
	*Probes:* What made your health care experience easier? Harder?
2	What have been your experiences with care related to prevention and/or management of *secondary complications*?
	*Probes:* What made it easier? Harder?
3	What has been the role of your informal social networks (friends/family) related to secondary complications?
8	Is there anything else you would like to mention that we have not had the opportunity to discuss?

*Additional probes were used to facilitate discussion if needed such as “Can you tell me more about that? Can you speak more about the process? How so?”

#### Quantitative measures

##### Population characteristics: demographics and clinical characteristics

Demographic items included the following: age, sex, education, income, ethnicity, language, occupation, employment status, marital status, area of residence (urban/rural), and insurance source for medical care. Clinical characteristics included items such as level of injury, mechanism of injury, and date of injury.

##### Social networks: formal and informal care networks

Formal care networks (e.g. physicians, rehabilitation professionals, and alternative medicine providers) were assessed using items from the CCHS.^47^ For example, “Not counting when you were an overnight patient, how many times have you seen or talked on the telephone, about your physical, emotional or mental health with… a family doctor…a specialist… a nurse…a physical therapist…a psychologist…” Participants were asked to provide the initials of the care provider.

The ASSIS is a semi-structured tool that consists of a series of questions that asks about a person's perception of network size and the adequacy of the support received (i.e. satisfaction and need). In particular, the ASSIS measures six functional areas of social networks: (1) material aid; (2) physical assistance; (3) intimate/private interaction; (4) guidance; (5) feedback, and (6) negative social interaction. This tool allows for the following network properties to be measured: (1) network size (including available and utilized social networks), (2) network composition, (3) support satisfaction, (4) support need, and (5) any sources of network conflict.^49^ The ASSIS has shown good test–retest reliability for size of the available network with correlations ranging from 0.70 (over 1-month period) to 0.88 (over 2 or more days).^49,51^

The survey starts with the following text: “In the next few minutes I would like to get an idea of the people who are important to you in a number of different ways. I will be reading descriptions of ways that people are often important to us. After I read each description, I will be asking you to give me the first names, the initials, or nicknames of the people who fit the description. These people might be friends, family members, teachers, priests, ministers, doctors, or other people who you might know. If you have any questions about the descriptions I have read, please ask me to try to make it clearer.”

For each functional area, the following related to (1) size, (2) satisfaction, and (3) need were asked of a participant. For example, for the intimate interaction domain: (1) Size –“If you wanted to talk to someone about the things that are very personal and private, who would you talk to? Give me the first names, initials, or nicknames of people who you would talk to about things that are very personal and private”, (2) Satisfaction – “How would you rate your satisfaction or dissatisfaction with the times you talked to people about your personal and private feelings during the past month?” (Response options include very dissatisfied, moderately dissatisfied, slightly dissatisfied, neither satisfied nor dissatisfied, slightly satisfied, moderately satisfied, or very satisfied.), and (3) Need – “During the past month, how much do you think you needed people to talk to about things that were very personal and private? Tell me which statement best describes your need, no need at all, slight need, moderate need, great need, or very great need.”

##### Secondary health conditions

The SCI-SCS-Modified is a 23-item measure of SHCs that impact health and physical functioning.^50^ Modified from the longer 40-item Secondary Complication Questionnaire (SCQ), the SCI-SCS was designed to measure complications related to skin, musculoskeletal, pain, and bowel/bladder in the past year. The measure uses a 4-point ordinal scale ranging from 0 (not experienced/insignificant problem never limiting activity) to 3 (significant/chronic problem). Items are summed up and scores can range from 0 to 69, with the higher score reflecting greater problems with SHCs. The SCI-SCS has shown good convergent validity, internal consistency (>0.80), and test–retest reliability (>0.60).^50^

##### Data analysis

All key informant interviews were audio-recorded and transcribed verbatim. Data analysis used an iterative constant comparative process involving descriptive and interpretive analyses.^43,46,52^ Using a template analysis approach,^53^ a flexible coding structure was developed based on the NEM, which allowed for free nodes when emerging ideas or themes were identified. After each interview, the principal investigator (S.J.T.G.) wrote detailed reflexive notes on major emerging themes, which were later discussed in detail with one of the research investigators (S.B.J.). The principal investigator (S.J.T.G.) coded all transcribed interviews. The other investigators (S.B.J., L.L.-C., B.C.C., T.C., and M.A.M.) independently reviewed a sample (*n* = 3, 20%) and compared the emergent themes. Data management was facilitated using NVivo9 qualitative analysis computer software, as well as SPSS (SPSS Inc, Chicago, IL) Version 19 for descriptive quantitative analyses.

## Results

Fourteen key informant interviews were conducted (6 men and 8 women). Demographics of the sample are shown in Table 2. The median age was 47.5 years (31–75). The median number of years post injury was 18 (range of 4–49 years). Approximately half of the participants had an injury at the level of the cervical spine (*n* = 8). For mobility aids, approximately half of the participants used electrical wheelchairs (*n* = 8), and the others used manual wheelchairs (*n* = 6). Approximately a third of the participants had an education level of high school or less (*n* = 4), associate's degree or bachelor's degree (*n* = 5), or graduate-level degree (*n* = 5). Eight individuals lived with a spouse and/or common-law partner, one individual lived with a paid attendant, and five people lived alone.

**Table 2:** Demographics of participants (*n* = 14)

Demographic/clinical characteristic	*n*
Mechanism of injury	
Traumatic SCI-motor vehicle related	4
Traumatic SCI-non motor vehicle related	7
Non-traumatic SCI	3
Level of injury	
Cervical	8
Thoracic	5
Lumbar	1
Injury severity	
Tetraplegia	8
Paraplegia	6
Relationship status	
Single/divorced	4
Married/common-law	8
Dating	2
Living status	
Alone	5
Spouse/partner	8
Paid attendant	1
Education level	
Less than high school	1
High school	3
Associates degree/bachelor degree	5
Master's degree	4
Doctorate degree	1
Family income (includes spouse if applicable)	
Under 29 000	3
60–69 000	1
> 100 000	5
Declined	3
Insurance funding	
Motor vehicle insurance	2
Public disability support	8
None	4
Employment status	
Return to work – yes	7
Return to work – no *	7

*Reasons for not returning to work (*n* = 7): six due to SHCs, one individual took early retirement.

The majority of individuals (*n* = 13) reported significant challenges with SHCs in the past year. In particular, significant or chronic problems were related to pressure sores, muscle spasms, and pain. UTIs were experienced in the last year for eight participants, five of whom reported these infections to be moderate to significant problems. The mean score on the SCI-SCS was 15.3 (SD = 8.2).

### Composition of social networks

#### Overall network size

Using both the CCHS and ASSIS tools, the total network size was calculated. The median network size for participants was 16.5 (range 5–28), which includes family, friends, and health care providers. Table 3 shows the composition of networks by gender. Similar network compositions were identified for both males and females, with the exception of females having more friends comprising the informal networks (median = 7.0 for females versus 4.5 for males).

**Table 3:** Identified informal and formal networks by composition, family, friends, and health care providers (*n* = 14)

Median number of individuals				
Gender	Family	Friends	Health Care Providers	Overall
Male participants (*n* = 6)	4.5	4.5	6.5	16.0
Female participants (*n* = 8)	4.5	7.0	6.0	17.0
Overall	4.5	5.5	6.0	16.5

#### Informal networks

Participants are encouraged in the ASSIS to identify any formal and informal members that fit within the six domains. However, with the exception of four participants, *individuals only identified family and/or friends* rather than formal health care providers within their social networks for the six functional areas within the ASSIS. Specifically, only three individuals identified paid health care professionals as members of their networks within the functional areas of intimate/private as well as advice. All other participants only identified informal care providers. Data analyses were conducted with both formal and informal networks; however, given the small number of formal health care providers identified in the ASSIS, the median values remained unchanged. Therefore, the following results will refer to *only informal care providers* (family and friends) identified using the ASSIS.

### Informal available networks

The overall median for available informal networks was 11.0 (range 3–19). Networks were larger for social support (median = 6.5) and physical assistance (median = 4.0), followed by positive feedback (median = 3.5), advice (median = 3.0), material assistance (median = 2.5), and intimate relations (median = 2.5; see Table 4).

**Table 4:** Informal network composition, satisfaction, and need, based on the six ASSIS domains

	Available*	Used*	Satisfaction^†^	Need^‡^
Intimate	2.5	1.0	7.0	3.0
Material	2.5	0.0	7.0^§^	1.0
Advice	3.0	1.5	7.0	2.0
Positive feedback	3.5	2.0	7.0	1.4
Physical assistance	4.0	2.5	7.0	5.0
Social support	6.5	5.5	7.0	5.0
Negative feedback	0.0	0.0		

*Median values are reported due to small numbers.

^†^Ordinal scale, 1 = very dissatisfied to 7 = very satisfied.

^‡^Ordinal scale, 1 = no need to 5 = very great need.

^§^Only one person reported using material assistance.

### Informal utilized networks

The size of the utilized networks was considerably smaller than the available networks. Utilized network medians were largest for social support (median = 5.5) and physical assistance (median = 2.5). Only one participant reported using material assistance, the remaining sample did not use material support (median = 0.0).

### Perceived satisfaction and need

In all six functional areas, participants reported being very satisfied with their networks (medians = 7.0; see Table 4). There was slightly more variation in perceptions of need, as median scores ranged from 1.0 (no need for material assistance) to 5.0 (great need for social support and physical assistance).

#### Shifting networks following SCI

The majority of individuals felt that their social networks decreased since their SCI, three individuals (21.4%) reported networks were moderately to significantly decreased, and four individuals (28.6%) felt that the networks slightly decreased. Four individuals (28.6%) stated that their networks did not substantially change.

### Thematic results: informal social network roles

**Figure 2:**
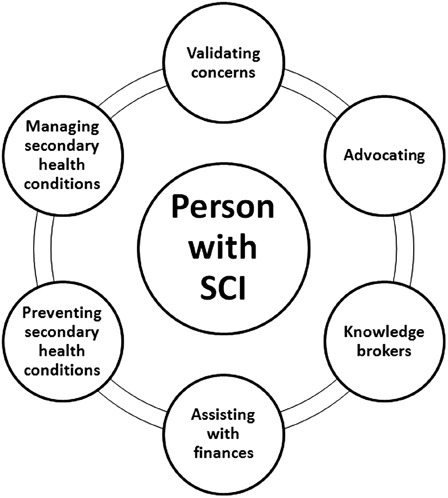
Thematic results

Numerous roles of informal networks were identified related to SHCs (see Fig. 2).

### Advice/validating concerns

Participants described how informal networks served as resources for advice related to SHCs, particularly with respect to validating concerns. Participants often spoke of uncertainty regarding the severity of a SHC and whether or not formal medical assessment/treatment would be warranted. In particular, informal networks often assisted individuals in clinical decision making of and self-management of SHCs.

They're the ones [wife and son] that sort of see me regularly and a lot of times I'll just sort of go is it me or is it really a problem… So if there's ever anything that's sort of a concern or bothering me, I always sort of go to her [wife] first just because I know if I'm at the point where I'm squawking about something, there's something wrong. (Interview Male 011)

### Knowledge brokers

Participants described their informal networks as playing an integral role in the acquisition of knowledge related to prevention of and/or management of SHCs. Informal networks assisted individuals with researching and acquiring clinical information, seeking appropriate health services, as well as facilitating knowledge exchange with various health care providers. Furthermore, participants described how their informal networks acted as key players in facilitating linkages to health care professionals with appropriate expertise in managing SHCs.

It was just by luck my wife knew a nurse through a friend of ours who was a bed sore nurse that travelled the world. She said “let me take a look at the wound.” She took a look at the wound. She offered this new product… and it cured me in 6 weeks. The doctor didn't even know about this and he wanted to put me in the hospital for a month after the operation. (Interview Male 013)

In particular, the CPA-Ontario division was mentioned by several participants as being a key *lifeline* for knowledge brokerage. For example, participants described how the CPA-Ontario provided individuals with a wide range of important information both directly and indirectly related to SHCs, such as self-management, community re-integration (work and volunteer opportunities, and social opportunities), housing and personal support, community health resources, and funding support (assistive devices and equipment, and disability support).

Like I said, CPA, if it hadn't been for the regional coordinator, I would have been left slapping in the wind (Interview Female 008)

### Advocacy

Participants discussed how informal networks played a strong advocacy role, especially with respect to SHC management and ensuring that they received timely and appropriate care. Family members and friends often accompanied participants to medical appointments to assist with knowledge exchange and advocate if needed. Participants commonly reported a feeling that the medical community dismissed concerns related to SHCs and appreciated the advocacy role that informal networks played.

I had an encounter with an orthopod who totally dismissed… I had a low energy fracture of the left tibia several years ago. I rolled over in bed and broke it and the first ortho that I saw he says “there's no break there.” Meanwhile my leg is twice the size that it should be and bruised and hot and everything else. My sister actually pointed it out on the x-ray to him. (Interview Female 007)

### Assisting with finances

In addition, informal networks also assisted individuals in filling out lengthy documentation for equipment funding, such as financial support for pressure relief cushions, wheel chairs, and home and vehicle modifications. The assistance with these applications was noted to be critically important, as access to assistive devices such as pressure relief cushions was essential in minimizing the occurrence of pressure sores. Participants described that the applications for funding support were time consuming and complicated, and informal networks provided substantial support in negotiating these funding processes.

Another important aspect to financial assistance is the personal financial contribution that informal networks provided. Many individuals described the financial “sacrifice” that their informal networks made, particularly family members, in order to minimize the occurrence of SHCs.

I don't reuse catheters. We [wife and participant] made a conscientious decision when this happened not to reuse catheters. We have the additional cost. Catheters are about $1 a pop… and I go through about 4 or 5 a day. It is a big cost and it's not covered by insurance. But we said we will wear that cost not to go through the risk of urinary tract… But no issues that way but I think it's because we're being very proactive and a lot of people I know aren't in a position where they can afford to sort of buy these things on a one use basis. (Interview Male 011)

### Preventing SHCs

Participants were asked about their experiences with prevention and/or management of SHCs. With respect to facilitating prevention, participants described how informal networks assisted with important prevention behavior such as skin checks for pressure sores, swelling, bruises, etc.

I was sitting in a living room one day and I hadn't really noticed any difference in the swelling of my feet. But my boyfriend did. He's like “I don't like the way your feet are looking, they're really swollen for you.” I looked down and it's like oh yeah, I guess they are… So they can sometimes pick up things that you don't and I think that's extremely valuable… You get those that you trust to kind of do the areas that you can't see and they get to a point where they might see something that you're not aware of. (Interview Female 007)

### Managing SHCs

Participants indicated that informal networks provided significant assistance with managing SHCs. In addition, informal networks provide assistance in facilitating interaction with health care providers such as setting up medical appointments, transportation to and from medical appointments, as well as physical assistance in physically negotiating often poorly accessible medical offices/examination tables, as part of their role in managing SHCs.

Especially for me because my biggest handicap… I can't even push my hands to click a button or Bluetooth or anything. She [wife] has to do everything on the phone, answer it, deal with all the VHA [home health care] and CCAC [Community Care Access Centre, home care] and everything because it's useless to hand it to me because she's going to do all the follow up. (Interview Male 013)

Informal networks provided substantial personal care with daily secondary complication management such as bladder and bowel care.

… bowel regularity… My body works fine but it doesn't work on a regular enough cycle…I always have to have somebody with me, either it's to pull my pants down or to help maneuver the commode chair because it's a little awkward. So I fundamentally have to work my bowel schedule around when I have somebody in the house to help me. So what I do is I now take basically it's a suppository every two days and I have enough sensation I can tell if my bowels are getting full. So I can tell if there's pressure… and literally I almost run my life around the bowel schedule…the two people that have to handle this with me is either my wife or my son. So I literally have to sort of okay what's your schedule, where are you going, are you going to be in a meeting, just so I can make sure okay… it literally, it's day one or it's day two. If it's day two, you come home. Now my wife fortunately works literally 10 minutes down the road from the house, so if all of a sudden I'm going I've got to go now, I often can just pick up the phone and say please come home now if you can. And she does… (Interview Male 011)

### Quality of informal network relationships

Participants discussed the importance of their informal networks and the value that they placed on these relationships. Trust and flexibility were important characteristics of the relationships with informal networks. With respect to trust, individuals described how informal networks provided support, both emotional and physical, in a safe environment.

I think that you know a secondary team like the family and friends are just as important if not more so in some aspects of primary health care because the doctors, they see you briefly, they make the diagnosis. But then it's the secondary team if you will that do the day to day things… You're calling them at 3 am going help because you can't get cleaned up or things like that and I think the secondary team is definitely not given enough credit… you've got the system and the team… you know, these are people that you trust that will go to bat for you, that will speak up for you and yet will allow you to be vulnerable. They just take what comes with the disability as it comes… they are a secondary team because they're the ones that are doing the bowel cleanup, the catheters in the middle of the night, the dressing changes, skin checks. You see them every day whereas your healthcare practitioner you're lucky if you see once every 3 months, something like that. (Interview Female 007)

Participants described how the informal networks, in particular family members, always were on-call, available, and adaptable in dealing with issues related to SHCs. These informal networks were described as members of a “secondary team”, critical in the prevention and management of SHCs. Finally, individuals described informal networks changing following their injury, usually becoming smaller but stronger networks.

I found out who my friends were and who weren't my friends or were just acquaintances. I think my network has become a closer network to me, a smaller group of people but closer. (Interview Female 008)

## Discussion

### Size of caregiving networks

In this descriptive mixed-methods study, we examined the structure, role, and quality of networks of care for persons with an SCI using the NEM. We identified that individuals had an overall median informal and formal network size of 16.5 persons (range 5–28), which included health care professionals, community organizations, friends, and family members. In comparison with the general population, we identified that the size of overall networks for persons with SCI is slightly smaller, as networks in the general population range from 20 to 30 persons.^54–56^ However, these results are similar to other studies that have used the ASSIS as a measure of networks for other vulnerable populations with disabilities. Previous studies have identified networks to be 11.5 persons for both the mental health population^55^ and multiple sclerosis population.^57^ In the rheumatoid arthritis population, using the Social Network Delineation Questionnaire, Fyrand *et al.*^58^ identified a total network size of 15.8 persons. In the general population, size of networks is important, as larger a social network, the more likely information will be passed on and new contacts made.^59,60^

Using the ASSIS, we identified the median number of available informal networks to be 11 persons (range 3–19). These findings are similar to a previous study that identified the available social support networks for persons with SCI 2 years following injury to be approximately 8.3 persons.^61^ It is worth noting that the Social Support Questionnaire^62^ was used to measure social networks and this instrument has a ceiling of nine persons that can be identified. In our present study we did not have a ceiling limit, as the ASSIS has no restriction on the number of reported networks.

Specifically within intimate relations, participants had a median of 2.5 network members, which is smaller than the general population, as recently, Wellman *et al.*^63^ identified in the “Connected Lives Study”, persons living in Toronto felt “very close” to 4.1 network members. Social support and physical assistance networks were larger in size compared with the other four domains, and participants rated these two domains in particular to be of “very great need”. Participants also reported using these social support and physical assistance networks more than the other domains.

### Role and quality of relationships

In addition to network size, we examined the role of network members as well as the quality of these relationships. This present study highlighted the importance of understanding the qualitative nature of social networks and the roles which individuals play within the context of SHCs. While the size of the informal networks may be smaller than that of the general population, the close ties with informal networks described by participants in the qualitative interviews was evident. Specifically, bonding cohesive social capital, characterized by intimate relationships in which social and psychological support are provided to help with day to day care needs^64^ was prominent among participants rather than bridging social capital. Bonding social capital is typically provided by relationships with family members as often these ties involve a significant amount of time with strong intimacy and trust.^65^ Key players within the social support and physical assistance networks were often family members, particularly spouses and/or significant partners of participants. The latter type of social capital, bridging, is based on weaker ties which are better suited to providing instrumental resources (e.g. access to community services and knowledge diffusion) rather than emotional or physical support.^59^

Previous research has identified the utility of bridging capital with the “strength of weak ties”^59^ and “structural holes”,^66^ that is, by increasing the number of non-redundant connections, individuals in theory have greater opportunity to access resources.^24,67^ However, in the present research, our qualitative data suggests that participants greatly valued a closer and stronger level of trust with their informal networks given the vulnerability of care provision potentially required. Participants described their informal networks as a “secondary team”, that is, a critical and essential force in dealing directly and indirectly with SHCs. The roles to which the secondary team members engaged in dealing with SHCs were identified as the following: (1) advice/validating concerns; (2) knowledge brokers; (3) advocacy; (4) assisting with finances; (5) preventing SHCs; and (6) managing SHCs. These results support the strength of cohesion,^68,69^ as strong ties are important when an individual is in a more vulnerable position and there is a need for trust and certainty.^68^ The roles identified of informal networks for persons with SCI are similar to informal care roles identified for persons with other chronic conditions.^70–74^ Recently, Essue and colleagues identified key roles in the self-management partnership for persons with complex chronic conditions (i.e. chronic heart failure, chronic obstructive pulmonary disease, and diabetes) to include home-helper, lifestyle coach, advocate, technical care manager, and health information interpreter.

This research suggests informal networks serve as essential key players in filling in the gaps that exist within the formal health care system. In particular, the CPA-Ontario was identified as an essential organization to bridge this gap, serving as a key *lifeline* for knowledge brokerage and advocacy. The CPA-Ontario is a not-for-profit organization that provides services in the areas of peer support, regional services coordinators, membership, employment services, advocacy, information services, and attendant services.^75^

Indeed, the gaps and barriers to health service delivery for those with complex chronic disabilities have been previously documented.^18^ Recently, Meade *et al.*^76^ specifically highlighted gaps in formal provider knowledge, provider–patient collaboration, quality of provider–patient communication, and discrimination for persons with SCI. Consistent with these findings, the present study identified that informal networks serve as central advocates, knowledge brokers, and validate secondary complication concerns for persons with SCI.

This research highlighted that these small cohesive networks of close ties are indispensable for persons with a SCI. The reliance on these small care-giving networks highlights the vulnerability and fragility of the informal networks. Persons with SCI might benefit from a more expansive network rich with weak ties to access novel information and diverse resources (e.g. how to prevent SHCs, new technologies, how to apply for equipment funding). Developing and maintaining a larger diverse network with weaker ties may be unattainable for most people with SCI due to the constraints their condition imposes on their social life. Thus, given the complexity of the condition, persons with SCI may only have the time and effort to focus on very close relationships.

Furthermore, despite the presence of these strong informal networks, the majority of individuals (*n* = 13) reported significant challenges with SHCs in the past year. This raises concern as to what extent would persons with SCI be able to deal with SHCs if these informal networks were not available and/or be able to engage in the roles described in this research. Based on the present findings, individuals would likely be struggling significantly more to prevent and/or manage SHCs if only relying on the formal health care system. Importantly, there is already significant strain on informal caregivers in assisting with supportive care for persons with SCI^77–79^ and this present research also highlights the extensive roles of informal care providers with SHCs. These extensive roles may create an inequality of exchange,^39^ as previous research has shown that the more instrumental, information, and emotional support needed from friends, the less satisfaction in social life has been reported by persons with SCI.^80^ Similarly, Essue *et al.*^72^ noted that the informal care-giving relationship can create a conflict between the care recipient and care providers. In Canada, there is a growing recognition of the importance of informal care provision in general, and the need to support the social, health and economic well-being of informal care providers.^81^ For example, informal care provision affects participation in the workforce and recently there have been policy recommendations for employers to offer flexibility and financial compensation for care provision (e.g. family leave).^81^ Given the critical roles assigned to informal care providers, it is pertinent that governmental organizations implement structures and policies that minimize the burden of care and ensure care competencies.

Similar to Canada, in Australia, there is a growing acknowledgement of the challenges in access to community-based care and the burden informal care places on care providers.^72^ For instance, to assess eligibility for community care services, the Commonwealth Government's Nationally Consistent Assessment includes “The Carer Eligibility and Needs Assessment-Revised”(CENA-R) questionnaire as one of the tools to measure carers' needs and the impact of caregiving on the relationship with the care recipient.^82^ In Canada, the home care assessment process might benefit from such a tool that involves the care provider characteristics, as well as relationship attributes in determining eligibility.

### Limitations of the present research

There are some limitations in this study. First, the ASSIS domains may have been too general for persons with SCI, given the complexity of the condition and the wide range of roles in which network members may serve. Given this, the qualitative data were critical to uncover the specific roles informal networks play in dealing with SHCs. For example, most participants did not recall any specific network member in assisting with material aid; however, in the qualitative interviews, findings suggest that informal networks serve a key role in assisting with financial costs. There were several similarities between domains in the ASSIS and those that were identified from the qualitative data such as advice, social support, positive feedback (validation of concerns); however, the qualitative data identified some new items particularly related to process facilitation roles with more specificity to SCI, such as knowledge brokerage, and assisting with funding applications for durable medical equipment and assistive devices.

Currently, there is no gold standard on measuring social support or social capital.^39^ Thus, the comparison of network size is challenged by the different measures used. Future research in developing metrics on social capital for persons with disabilities may be warranted to explore whether these are common roles for informal care providers assisting those with any complex physical disability. For example, items might address informal caregivers' time off work, costs associated with assistive devices, equipment, medical supplies, technology, transportation, and indirect health care costs not covered under public or private insurance plans. Understanding the role of informal care providers for any complex condition in more detail would be beneficial to policy planning. In addition, future research would be useful in understanding how individuals acquire the networks (e.g. passively assigned or actively sought) and how these network structures change over time.

Finally, another limitation is that these results are based on a small sample of individuals with SCI and need to be considered in other settings. Specifically, persons with high ventilation tetraplegia or individuals requiring 24-hour care are underrepresented. There was an explicit effort for balanced gender distribution for this initial exploratory understanding of social networks; however, this does not reflect the gender distribution of SCI.^3^

However, this research is useful as a foundation in understanding care networks and SHCs for persons with SCI. In using the NEM, understanding these networks of care and how they may relate to health care utilization and outcomes, recommendations to improve the management of care for the SCI population at the individual, provider, and policy level can be made. Future research is warranted in examining other components of the NEM, specifically the health care environment/system, and how the components of the model interact in the journey of care for persons with SCI. Our research highlighted that while networks are smaller for persons with SCI, these ties are stronger, which is essential when the roles involve a level of trust, certainty, tacit knowledge, and flexibility. Indeed, these networks serve as a critical secondary team for persons with SCI.
